# Correction: Environmental and climate variability drive population size of annual penaeid shrimp in a large lagoonal estuary

**DOI:** 10.1371/journal.pone.0306830

**Published:** 2024-07-05

**Authors:** The PLOS ONE Staff

## Notice of Republication

This article was republished on June 21, 2024, to correct errors in Figure 2. The publisher apologizes for the errors. Please download this article again to view the correct version. The originally published, uncorrected article and the republished, corrected articles are provided here for reference.

## Supporting information

S1 File Originally published, uncorrected article. (PDF)

S2 File Republished, corrected article. (PDF)
