# Supplementary figures and images for: Environmental and climate variability drive population size of annual penaeid shrimp in a large lagoonal estuary
Source: PLoS One. 2023 May 15;18(5):e0285498. doi: 10.1371/journal.pone.0285498 (PMC10184946; doi:10.1371/journal.pone.0285498)

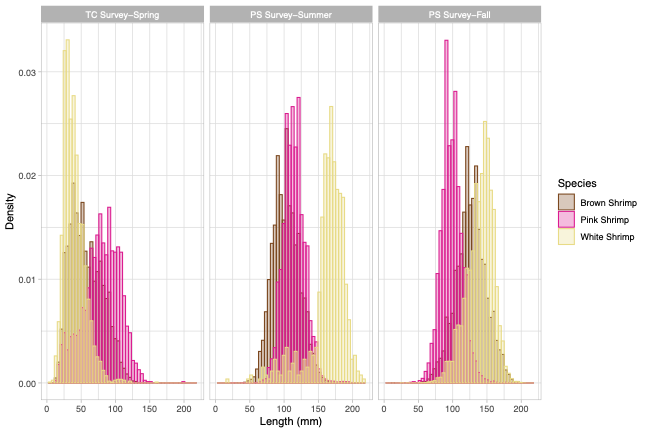

Supplement: S1 Fig — This figure represents length data from 94,952, 13,775, and 15,732 brown shrimp; 1,815, 10,740, and 11,025 pink shrimp; and 2,662, 607, and 17,300 white shrimp, for the spring P120, summer P195, and fall P195 trawl surveys, respectively. (TIFF) [file pone.0285498.s001.tiff]

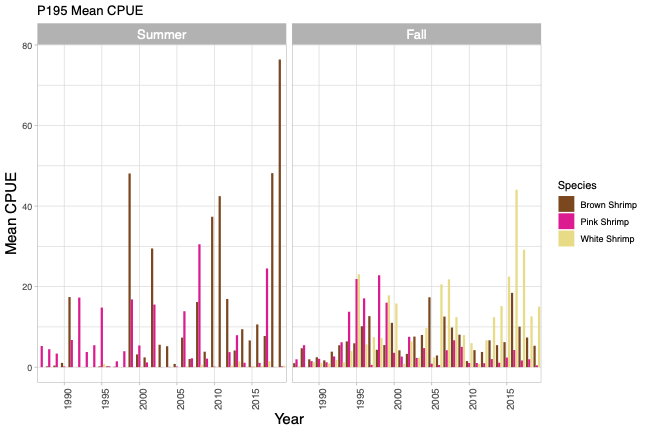

Supplement: S2 Fig — (TIFF) [file pone.0285498.s002.tiff]

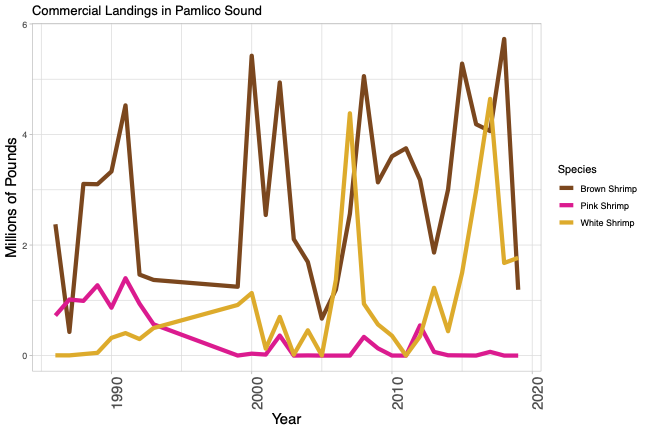

Supplement: S3 Fig — (TIFF) [file pone.0285498.s003.tiff]
