# Supplementary material for: Environmental and climate variability drive population size of annual penaeid shrimp in a large lagoonal estuary
Source: PLoS One. 2023 May 15;18(5):e0285498. doi: 10.1371/journal.pone.0285498 (PMC10184946; doi:10.1371/journal.pone.0285498)
Supplement: Attachment — Submitted filename: Response to Reviewers.docx [file pone.0285498.s005.docx]

**From Dr. Ottersen:**

**As the evaluations from the three reviewers show you are very nearly there. However, ref 3 points to one issue that you should look further into before I accept for publication. Further to that point you could check Stratton et al. 2019 Fisheries Oceanography.28:487–504. There they analyzed relative abundance of fish and shellfish species in SEAMAP data and found that in the southeastern US, “for the assemblage as a whole, fishing effects predominated over climate effects. In particular, changes in trawling effort (#trips) within the penaeid shrimp fishery governed abundance trends…changes in trawling effort within the penaeid shrimp fishery governed abundance trends of bony fishes, invertebrates, and elasmobranchs, a likely result of temporal changes in bycatch mortality.”**

Thank you Dr. Ottersen for your help in improving our manuscript. As both you and reviewer three noted, our models use commercial shrimp catch and the SEAMAP CPUE as abundance indicators of shrimp in Pamlico Sound, NC. We feel that this is a stronger approach than using commercial shrimp effort (i.e., number of trips). We are familiar with Dr. Stratton’s paper, and it is cited in our manuscript. Stratton et al. 2019 showed that many species of finfish, elasmobranchs, and invertebrates in the US Southeast were significantly affected by effort in the shrimp fishery. However, this is primarily due to the fact that these species are caught as bycatch in the shrimp fishery. Importantly, shrimp are not a bycatch species (i.e., they are not discarded) and in Stratton et al.’s paper, **shrimp abundance was not significantly related to commercial shrimping effort** (Stratton et al., 2019, Table 4). Stratton et al. also used effort data because there are no reported bycatch data, and presumably, if estimates of bycatch were available those data would have been an even better predictor than effort data for his study. This suggests to us that adding effort in our models would not improve our model fit. Additionally, a long-standing issue in North Carolina is that effort in the shrimp fishery is poorly resolved. Because shrimp are not a bycatch species in the shrimp fishery, we feel strongly that commercial catch data and the fisheries independent SEAMAP CPUE are much better indicators of abundance and our data support that these variables are good predictors of shrimp abundance. Our models resolve 37-81% (mean: 64%) of the variation in the data indicating that they do a very good job of describing the patterns in abundance data. Thank you again for your care and attention to our manuscript and for considering our response.

**Reviewer #3: I thank the authors for their thorough response to this lengthy set of reviews. However, my one, primary concern with the study remains. As I understand it, goal #1 of this study was to be able to model “the spatial distribution and abundance of sub-adult and adult penaeid shrimp”. I am still wondering if fishery effort (e.g., #trips) could be an even better explanatory variable for spatial patterns in adult shrimp abundance than catch.

The authors’ response indicated that commercial catch and SEAMAP CPUE were considered as factors in their models (e.g. Lines 150 and 164). But SEAMAP CPUE is an index of abundance and catch alone can be influenced by a lot of things (it’s often hard to say what it’s a measure of). The authors went on to say that “in this fishery, higher catch is proportional to overall greater abundance and spawning stock biomass”, which they have demonstrated. But can you better predict spatiotemporal patterns in abundance with effort than with catch, especially given shrimp trawl fishing effort has been shown to be highly influential (more so than environmental variables) in predicting SEAMAP relative abundance for many southeastern species due to the bycatch impacts of this fishery.**

Thank you for your help in improving our manuscript and for the thoroughness of your reviews. You are absolutely correct that shrimp trawl effort has been shown to be a very important predictor variable, even outweighing climate data, in an important study by Stratton et al. 2019. We are familiar with Dr. Stratton’s paper, and it is cited in our manuscript. However, for several reasons we feel strongly that commercial catch of shrimp and SEAMAP CPUE of shrimp are more appropriate predictor variables than commercial shrimp effort in our models examining the population trends of shrimp in Pamlico Sound, North Carolina.

As you note, we're using catch data, lagged one year, as an indicator of spawning stock abundance. This assumes that commercial catch might be related to abundance, which is highly variable from year to year. Because catch has biases associated with effort, we are also using SEAMAP indices as an independent abundance indicator. When included in the models (through model selection criteria), commercial catch was positively related to the following year's recruitment, on all accounts, suggesting that increased catch indicated higher abundance (see Figures 4 and 9). If fisheries effects were negatively impacting recruitment, we'd expect a negative relationship, which we do not see.

While a number of species examined in Stratton et al. 2019 were influenced by shrimp trawl effort, shrimp were not among these species. For species that did show such a relationship, it was primarily due to the fact that these were species caught as bycatch in the shrimp fishery. Importantly, shrimp are not a bycatch species (i.e., they are not discarded) and in Stratton et al.’s paper, **shrimp abundance was not significantly related to commercial shrimping effort** (Stratton et al., 2019, Table 4). Stratton et al. also used effort data because there are no reported bycatch data, and presumably, if estimates of bycatch were available those data would have been an even better predictor than effort data for his study. This suggests to us that adding effort in our models would not improve our model fit. Additionally, a long-standing issue in North Carolina is that effort in the shrimp fishery is poorly resolved.

Because shrimp are not a bycatch species in the shrimp fishery, we feel strongly that commercial catch data and the fisheries independent SEAMAP CPUE are superior indicators of abundance compared to effort. **Most importantly, our results show that both SEAMAP CPUE and commercial catch data are good predictors of shrimp abundance**. Our models resolve 37-81% (mean: 64%) of the variation in the data indicating that they do a very good job of describing the patterns in abundance data.

We are thankful to have had such attention to our manuscript from yourself and the other reviewers and are very appreciative of your time and effort. Thank you for helping us to improve our manuscript!
